# Medical News and Miscellany

**Published:** 1897-12

**Authors:** 


### Medical News and Miscellany.

**Dr. W. E. Magee** has located at Chilton, Falls County, Texas.

---

**Dr. Harrison Allen,** Emeritus Professor of Comparative Anatomy in the Medical Department of the University of Pennsylvania, died of heart disease on Nov. 14th.

---

**Good Country Practice,** with comfortable house, outbuildings, etc., for sale at a sacrifice. Good reasons for wanting to sell. A small cash payment and balance on time. Address Dr. G. M., care of TEXAS MEDICAL JOURNAL.

---

**Requiescat in Pace.**—The Medical Magazine of Atlanta, Ga., erstwhile "Moody's M. M." after a brief and fitful career under the editorial management of Dr. Ralcy H. Bell, sleeps with its forefathers. No, it had no forefathers, it was *sui generis*, a sport on a well-known species, and too "fit" to survive.

---

**The New York Polyclinic.**—We have for sale at a discount two orders for $50 each on this famous school. These orders can be presented and will be accepted in lieu of cash. To a physician who is going to take a post-graduate course during the coming fall or winter, this is a chance to save money. Address the TEXAS MEDICAL JOURNAL.

---

**Wanted.**—To purchase a practice in some railroad town in Texas. A good location off railroad not objected to. Please write describing your location, value of practice annually, and number of inhabitants in town. Would buy resident property to get good location. South Texas preferred. Address,

J. R. GEORGE, M. D.,
Corrigan, Texas.

**Change of Base** and hands-all-round: The *Courier Record of Medicine* has removed from Dallas to Fort Worth, Texas, where it was founded by the Senior Editor of this Journal some years ago and sold to Dr. Brooks. Dr. Brooks' widow and two sons publish the journal; while having discontinued its Dallas staff of editors, the editorial department is "voluntarily taken charge of" by the faculty of the Fort Worth Medical School, *i. e.* Drs. Adams, Thompson, Saunders, Grammon, Gray and Chase and others. We wish them well. We didn't know the tripod was so fascinating; nor how rich Texas is in embryo medical editors. The first issue (October) under the new management is very creditable.

